# The Long-Term Impact of Bariatric Surgery on Development of Atrial Fibrillation and Cardiovascular Events in Obese Patients: An Historical Cohort Study

**DOI:** 10.3389/fcvm.2021.647118

**Published:** 2021-04-13

**Authors:** Hongtao Yuan, Jose R. Medina-Inojosa, Francisco Lopez-Jimenez, William R. Miranda, Maria L. Collazo-Clavell, Michael G. Sarr, Alanna M. Chamberlain, David O. Hodge, Kent R. Bailey, Yutang Wang, Yundai Chen, Yong-Mei Cha

**Affiliations:** ^1^Department of Cardiology, Chinese People's Liberation Army General Hospital (PLAGH), Beijing, China; ^2^Department of Cardiovascular Medicine, Mayo Clinic, Rochester, MN, United States; ^3^Division of Endocrinology, Diabetes, Metabolism, and Nutrition, Mayo Clinic, Rochester, MN, United States; ^4^Department of Surgery, Mayo Clinic, Rochester, MN, United States; ^5^Department of Health Sciences Research, Mayo Clinic, Rochester, MN, United States; ^6^Department of Health Sciences Research, Mayo Clinic, Jacksonville, FL, United States

**Keywords:** atrial fibrillation, bariatric surgery, gastric bypass, obesity, Roux-en-Y

## Abstract

**Objective:** To determine whether early Roux-en-Y gastric bypass surgery (RYGB) reduces the risk of Major adverse cardiovascular events (MACE) in patients with obesity.

**Patients and Methods:** We conducted a study of patients with class II and III obesity [body mass index (BMI) > 35 kg/m^2^] from Olmsted County, Minnesota, who underwent obesity clinic consultation between the years 1993–2012, and had either RYGB surgery within 1 year (RYGB-1Y group), or medically managed (No-RYGB group). The composite endpoint of MACE (all-cause mortality, stroke, heart failure admission and acute myocardial infarction) was the primary endpoint, with new-onset AF as the secondary endpoint.

**Results:** Of the 1,009 study patients, 308 had RYGB-1Y and 701 were medically managed (No-RYGB). Overall, the age was 44.0 ± 12.4 (mean ± SD) years; BMI was 45.0 ± 6.8 kg/m^2^. The RYGB-1Y group had a lower rate of MACE (adjusted hazard ratio (HR), 0.62; 95% CI, 0.44–0.88; *P* = 0.008) and lower mortality (adjusted HR, 0.51; 95% CI, 0.26–0.96; *P* = 0.04) than the No-RYGB group. The RYGB-1Y surgery was not associated with lower AF occurrence (HR, 0.66; 95% CI, 0.40–1.10; *P* = 0.11).

**Conclusion:** An early RYGB approach for BMI reduction was associated with lower rates of MACE.

## Introduction

Obesity is a worldwide epidemic (1, 2), affecting more than one-third of adults in the United States (3). Obesity is associated with increased incidence of systemic diseases, such as insulin resistance, abnormal glucose metabolism, dyslipidemia, hypertension, obstructive sleep apnea (OSA) and coronary disease (4–10). In particular, obesity has been associated with a 2.4-fold increased atrial fibrillation (AF) risk in men and a 2-fold increased AF risk in women (11). AF is also often described as epidemic and is associated with multiple adverse sequelae, such as stroke, heart failure, dementia, diminished quality of life, and death (12). Although aging of the population is considered an important contributor to this epidemic, obesity and its associated cardiometabolic comorbidities may play a critical role.

Bariatric surgery is an effective approach in treating patients with morbid obesity, achieving long-lasting weight reduction and reducing concomitant comorbidities (13–15). A prospective randomized study showed that the use of Roux-en-Y gastric bypass (RYGB) in morbidly obese patients was associated with higher rates of diabetes remission and lower risk of cardiovascular events over a period of 6 years compared with non-surgical control patients (16). However, little is known about the long-term effect of RYGB on the development of AF or fatal and non-fatal cardiovascular events. The objective of this study was to determine whether early RYGB surgery reduces the incidence of AF or of major adverse cardiovascular events (MACE) in obese patients compared to nonsurgical therapy (diet, exercise, and medication) or late RYGB surgery.

## Materials and Methods

### Study Patients

We conducted an historical cohort study of 1,009 patients who received a diagnosis of class II-III obesity [body mass index (BMI) > 35 kg/m^2^] between January 1993 and December 2012 at Mayo Clinic (Rochester, MN), were residents of Olmsted County, MN, underwent an obesity clinic consultation and had either Roux-en-Y Gastric Bypass (RYGB) or medical management of obesity. We excluded patients who underwent gastric banding (*n* = 25) and those with incomplete data (loss of follow-up, *n* = 98). This study included only patients who had given research authorization and was approved by the Institutional Review Board.

### Obesity Clinic Consultation

For the current study, the index date was defined as the initial obesity clinic consultation. In our institution, the obesity clinic follows a standardized clinical protocol: (1) An initial clinical evaluation led by an endocrinologist, with the objective of identifying medical complications of obesity and surgical risks. (2) A dietitian assessment which reviews their eating habits and advises on dietary modifications and calorie restriction. (3) A behavioral psychologist assessed any cognitive impairment/untreated psychiatric disorders and develops behavioral therapy program. (4) Follow-up visit within 12–16 weeks of initial assessment, to evaluate the progress on weight maintenance, activity program, completion of behavioral therapy and treatment of medical conditions. (5) Surgical consultation generally occurs within 3–4 months after initial visit in the uncomplicated patient. The average duration from initial visit to the date of surgery in our practice is 8 months.

### Roux-en-Y Gastric Bypass

During the study period, patients underwent either open or laparoscopic RYGB surgery. These procedures divided the stomach into a small pouch (<50 mL) of proximal stomach that drained into a Roux-en-Y limb of jejunum. During the study period, the usual length of the Roux limb was 150 cm. The biliopancreatic digestive juices from the bypassed distal stomach and duodenum emptied in the mid jejunum leading to digestion and absorption of nutrients.

### Patient Clinical Information and Outcomes

Patient clinical information was collected from the electronic medical record and included, blood pressure (systolic and diastolic), BMI, history of coronary heart diseases, hypertension, or diabetes mellitus, fasting blood glucose level, creatinine level, total triglycerides, high-density lipoprotein cholesterol, and low-density lipoprotein cholesterol levels, and echocardiographic parameters were collected as of the index date. Risk of thromboembolic stroke associated with AF was calculated as the CHADS2 (congestive heart failure, hypertension, age, diabetes mellitus, prior stroke or transient ischemic attack) score. Information on outpatient prescriptions was collected electronically.

Any new-onset AF documented in the 12-lead ECG or Holter monitor was considered as the primary outcome; AF episodes occurring within 30 days of RYGB were considered to be post-operative and therefore not included in the analysis. The secondary composite endpoint consisted of MACE (all-cause mortality, stroke, heart failure admission and acute myocardial infarction) was also assessed. Clinical and outcome information was reviewed manually by two independent investigators; disagreement was resolved by consensus (*k* = 0.87). Last follow-up date was defined as the last clinic visit in each patient or December 31 2015.

### Statistical Analysis

We categorized those that underwent RYGB surgery within 1 year of initial consultation and were considered as the “early” surgical group (RYGB-1Y group), while the remaining patients were included as controls (No-RYGB-1Y group). The latter group included patients who received medical therapy alone and “late” RYGB surgery. Endpoints occurring within 1 year resulted in excluding the patient from this analysis. Comparisons within groups between baseline and follow-up were performed using paired *t*-tests. Comparisons between groups were performed using two-sample *t*-tests and Chi-square tests for continuous and categorical variables, respectively. Continuous variables are presented as mean ± standard deviation and categorical variables are presented as counts (%). The cumulative probability of freedom from AF and from secondary end-points were estimated using the Kaplan-Meier method. Potential risk factors for each of these endpoints were evaluated using Cox proportional hazards models. In order to evaluate the overall effect of surgery while taking into account all patients in the population, a supplementary analysis using surgery as a time dependent treatment variable was performed. In this analysis, patients were analyzed as medically treated until they received surgery and then considered as surgically treated, which enabled all events after the index obesity/nutrition visit to be analyzed.

## Results

### Patient Characteristics and Factors Related to Surgery

A total of 1,009 patients were included in the study, of these 544 had RYGB within the study period, 308 were included in the RYGB-1Y and 701 in the No-RYGB-1Y group. The mean (SD) age was 42.2 (12.0) years and 78% were females. Baseline characteristics are presented in Table 1. Hypertension, dyslipidemia, diabetes mellitus, coronary disease, heart failure and high CHADS2 score were the most common comorbidities and were associated with less likelihood of receiving early RYGB surgery within 1 year. There was no association between left ventricular ejection fraction or medication use and the likelihood of undergoing early bariatric surgery. The RYGB-1Y group had a higher BMI, and a lower systolic BP, glucose, HbA1c and creatinine level than No-RYGB-1Y patients. The No-RYGB-1Y group where more often prescribed statins, ACEI/ARB, beta blocker or calcium channel blockers, and insulin than the RYGB-1Y group.

**Table 1:** Baseline patient characteristics.

**Characteristics^a^**	**RYGB-1Y (*N* = 308)**	**No-RYGB-1Y (*N* = 701)**	***P-*value**
Age, y	44.2 (10.5)	43.6 (12.6)	0.17
Female, No. (%)	254 (82.5)	537 (76.6)	0.04
BMI, kg/m^2^	46.4 (6.5)	44.8 (6.9)	<0.001
**Underlying diseases, no. (%)**			
HTN	136 (44.2)	393 (56.1)	<0.001
DM	65 (21.1)	271 (38.7)	<0.001
Hyperlipidemia	134 (43.5)	372 (53.1)	0.005
OSA	87 (28.2)	199 (28.4)	0.96
CAD	15 (4.9)	64 (9.1)	0.02
Valvular heart disease	0 (0.0%)	3 (0.4)	0.25
Cardiomyopathy	0 (0.0%)	4 (0.6)	0.18
CKD	8 (2.6)	38 (5.4)	0.05
Stroke	3 (1.0)	4 (0.6)	0.48
CHF	1 (0.3)	25 (3.6)	0.003
**CHADS**_2_ **score, no. (%)**			<0.001
0	155 (50.3)	242 (34.5)	
1	103 (33.4)	233 (33.2)	
≥2	45 (14.6)	184 (26.2)	
Systolic BP, mm Hg	131.6 (15.1)	135.2 (14.2)	<0.001
Diastolic BP, mm Hg	77.6 (9.2)	79.3 (10.0)	0.006
LVEF, %	62.9 (6.7)	62.1 (8.4)	0.66
Hemoglobin, g/dL	14.0 (7.0)	13.5 (1.3)	0.25
HbA1c, %	5.7 (1.2)	6.3 (2.8)	<0.001
Cr, mg/dL	0.9 (0.2)	1.0 (0.2)	0.04
TG, mg/dL	193.1 (110.0)	181.5 (99.4)	0.13
HDL-C, mg/dL	46.4 (11.9)	46.0 (11.9)	0.69
LDL-C, mg/dL	120.1 (32.3)	118.4 (34.8)	0.24
**Medical therapy, no. (%)**			
Statins	49 (15.9)	211 (30.1)	<0.001
ACEI/ARB	50 (16.2)	190 (27.1)	<0.001
CCB	12 (3.9)	64 (9.1)	0.004
BB	31 (10.1)	114 (16.3)	0.01
Diuretics	53 (17.2)	148 (21.1)	0.15
Insulin	7 (2.3)	43 (6.1)	0.009
Metformin	3 (1.0)	73 (10.4)	<0.001
Aspirin	42 (13.6)	181 (25.8)	<0.001
Clopidogrel	2 (0.6)	10 (1.4)	0.29

*ACEI, angiotensin-converting enzyme inhibitor; ARB, angiotensin receptor blocker; BB, β-blocker; BMI, body mass index; BP, blood pressure; CAD, coronary artery disease; CCB, calcium channel blocker; CHADS2, congestive heart failure, hypertension, age ≥ 75 y, diabetes mellitus, prior stroke or transient ischemic attack; CHF, congestive heart failure; CKD, chronic kidney disease; Cr, creatinine; DM, diabetes mellitus; FBG, fasting blood glucose; HbA1c, hemoglobin A1c; HDL-C, high-density lipoprotein cholesterol; HR, hazard ratio; HTN, hypertension; LDL-C, low-density lipoprotein cholesterol; LVEF, left ventricular ejection fraction; OSAS, obstructive sleep apnea syndrome; TG, triglyceride*.

a *Values represent mean (SD) unless otherwise specified*.

### RYGB Surgery and Improvement in Metabolic Profile

BMI, systolic and diastolic blood pressure, triglycerides and LDL were reduced, and HDL was increased in both RYGB-1Y group and No-RYGB-1Y group at follow-up. The magnitude of changes, however, was greater in the RYGB-1Y group compared to No-RYGB-1Y group (Table 2). The hemoglobin A1c level was significantly decreased in the RYGB-1Y group while increased in the No-RYGB-1Y group.

**Table 2:** Comparison before and after therapy in RYGB-1Y group and No-RYGB-1Y groups.

**Characteristic**	**RYGB-1Y group (*N* = 308)**				**No-RYGB-1Y group (*N* = 701)**			
	**Before^a^**	**After**	**Delta**	***P*-value**	**Before**	**After**	**Delta**	***P*-value**
BMI, kg/m^2^	46.4 (6.5)	34.5 (7.4)	−12.3^*^ (6.6)	<0.001	44.8 (6.9)	41.1 (9.0)	−4.4 (9.2)	<0.001
Systolic BP, mmHg	135 (14)	121 (14)	−14^*^ (17)	<0.001	132 (15)	130 (20)	−2 (23)	0.004
Diastolic BP, mmHg	78 (9)	72 (10)	−6^*^ (11)	<0.001	79 (10)	77 (11)	−3 (13)	<0.001
Glucose, mg/dL	122 (43)	98 (21)	−24^*^ (36)	<0.001	117 (44)	115 (42)	−1 (50)	0.48
HbA1c, %	5.7 (1.2)	5.5 (1.0)	−0.2^*^ (1.0)	0.001	6.3 (2.8)	6.5 (3.1)	0.2 (1.5)	0.005
Cr, mg/dL	0.9 (0.2)	0.8 (0.3)	−0.1^*^ (0.2)	<0.001	1.0 (0.2)	0.9 (0.4)	−0.06 (0.35)	<0.001
TG, mg/dL	193 (110)	108 (60)	−84^*^ (91)	<0.001	182 (99)	149 (90)	−32 (96)	<0.001
HDL-C, mg/dL	46 (12)	61 (17)	14^*^ (15)	<0.001	46 (12)	52 (17)	6 (15)	<0.001
LDL-C, mg/dL	120 (32)	86 (26)	−35^*^ (31)	<0.001	118 (35)	94 (33)	−25 (38)	<0.001

*BMI, body mass index; BP, blood pressure; Cr, creatinine; HbA1c, hemoglobin A1c; HDL-C, high-density lipoprotein cholesterol; LDL-C, low-density lipoprotein cholesterol; TG, triglyceride*.

a *Before and after values represent mean (SD)*.

* *p < 0.05 comparing the delta's between the surgery and non-surgery groups*.

### Incidence of AF

**Figure 1:**
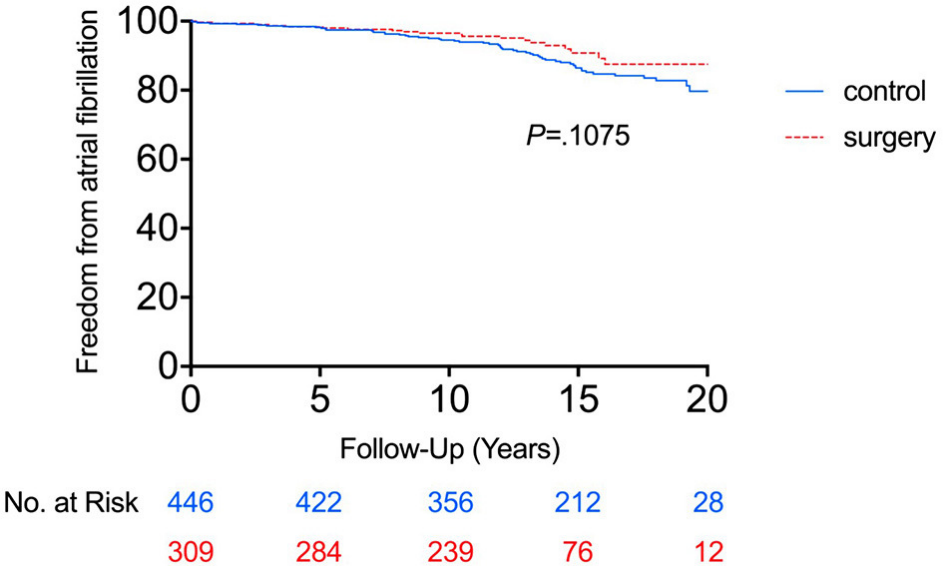
Freedom from atrial fibrillation between RYGB-1Y group and NO-RYGB group. Kaplan-Meier curve showed there was no significant difference in atrial fibrillation occurrence in obese patients treated with early RYGB surgery within 1 year of index diagnosis (RYGB-1Y) compared with obese patients treated with medical only (No-RYGB surgery).

Comparison of the cumulative incidence of AF between the RYGB-1Y and No-RYGB-1Y patients is provided in Figure 1. There was no significant difference between two groups (*p* = 0.22). After adjustment for age, sex, and baseline hypertension, diabetes, and BMI, there was no significant difference between surgery within 1 year and the rest of the group [HR = 0.91, 95% (0.43–1.90) *p* = 0.80]. Multivariate analysis showed that baseline BMI and CHADS2 score were independent factors associated with AF occurrence (Table 3).

**Table 3:** Univariate and multivariate analysis on atrial fibrillation occurrence.

	**Univariate**			**Multivariate analysis**		
**Variable**	**HR**	**95% CI**	***P*-value**	**HR**	**95% CI**	***P*-value**
Age	1.09	1.07–1.11	<0.001			
Female sex	0.50	0.29–0.87	0.01			
RYGB Surgery	0.64	0.31–1.31	0.22			
Baseline BMI	1.04	1.00–1.07	0.04	1.05	1.01–1.09	0.004
DM	2.95	1.72–5.05	<0.001	0.91	0.45–1.85	0.8
HTN	3.45	1.74–6.84	<0.001	0.76	0.31–1.83	0.5
OSA	2.85	1.69–4.79	<0.001	1.51	0.84–2.71	0.1
CHADS_2_ score	2.12	1.72–2.60	<0.001	2.20	1.56–3.12	<0.0001
Hyperlipidemia	1.99	1.15–3.46	0.01			
CAD	2.98	1.64–5.42	<0.001			
Cardiomyopathy	12.96	3.12–53.79	<0.001			
CKD	6.04	3.24–11.25	<0.001			
Stroke	1.11	0.41–3.02	0.83			
CHF	9.49	5.18–17.41	<0.001			
Statins	1.75	1.04–2.95	0.04			
ACEI/ARB	1.04	0.59–1.81	0.90			
CCB	1.46	0.71–3.01	0.03			
BB	1.43	0.78–2.63	0.25			
Diuretics	2.25	1.32–3.83	0.003			
Insulin	3.60	1.86–6.99	<0.001			
Metformin	0.94	0.37–2.35	0.89			
Aspirin	1.45	0.84–2.50	0.19			
Digoxin	44.25	13.31–147.11	<0.001			
FBG	1.01	1.00–1.01	0.01			
Cr	2.23	0.60–8.30	0.23			
TG	1.0	0.99–1.01	0.39			
HDL-C	0.99	0.97–1.01	0.33			
LDL-C	0.99	0.99–1.00	0.38			

*ACEI, angiotensin-converting enzyme inhibitor; ARB, angiotensin receptor blocker; BB, β-blocker; BMI, body mass index; CAD, coronary artery disease; CCB, calcium channel blocker; CHADS2, congestive heart failure, hypertension, diabetes mellitus, prior stroke or transient ischemic attack; CHF, congestive heart failure; CKD, chronic kidney disease; Cr, creatinine; DM, diabetes mellitus; FBG, fasting blood glucose; HDL-C, high-density lipoprotein cholesterol; HR, hazard ratio; HTN, hypertension; LDL-C, low-density lipoprotein cholesterol; OSAS, obstructive sleep apnea syndrome; TG, triglyceride*.

Supplementary analysis including surgery as a time dependent yielded similar result. This incorporated all of the 544 subjects who eventually had surgery. The result of this analysis was very similar to the primary analysis. Undergoing surgery was not associated with the development of AF [HR = 0.80 (95% CI 0.46, 1.41), *P* = 0.44].

Using the primary analysis curve in Figure 1, the cumulative probability of AF incidence at 15 years was 23.1% (95% CI 12.7–33.5%) in the No-RYGB-1Y group and 8.2% (95% CI 1.8–14.6%) in the RYGB-1Y group. If we do a crude point estimate comparison at 15 years of the rates there is a nominally significant difference between the 2 groups (*p* = 0.02).

### Incidence of MACE

**Figure 2:**
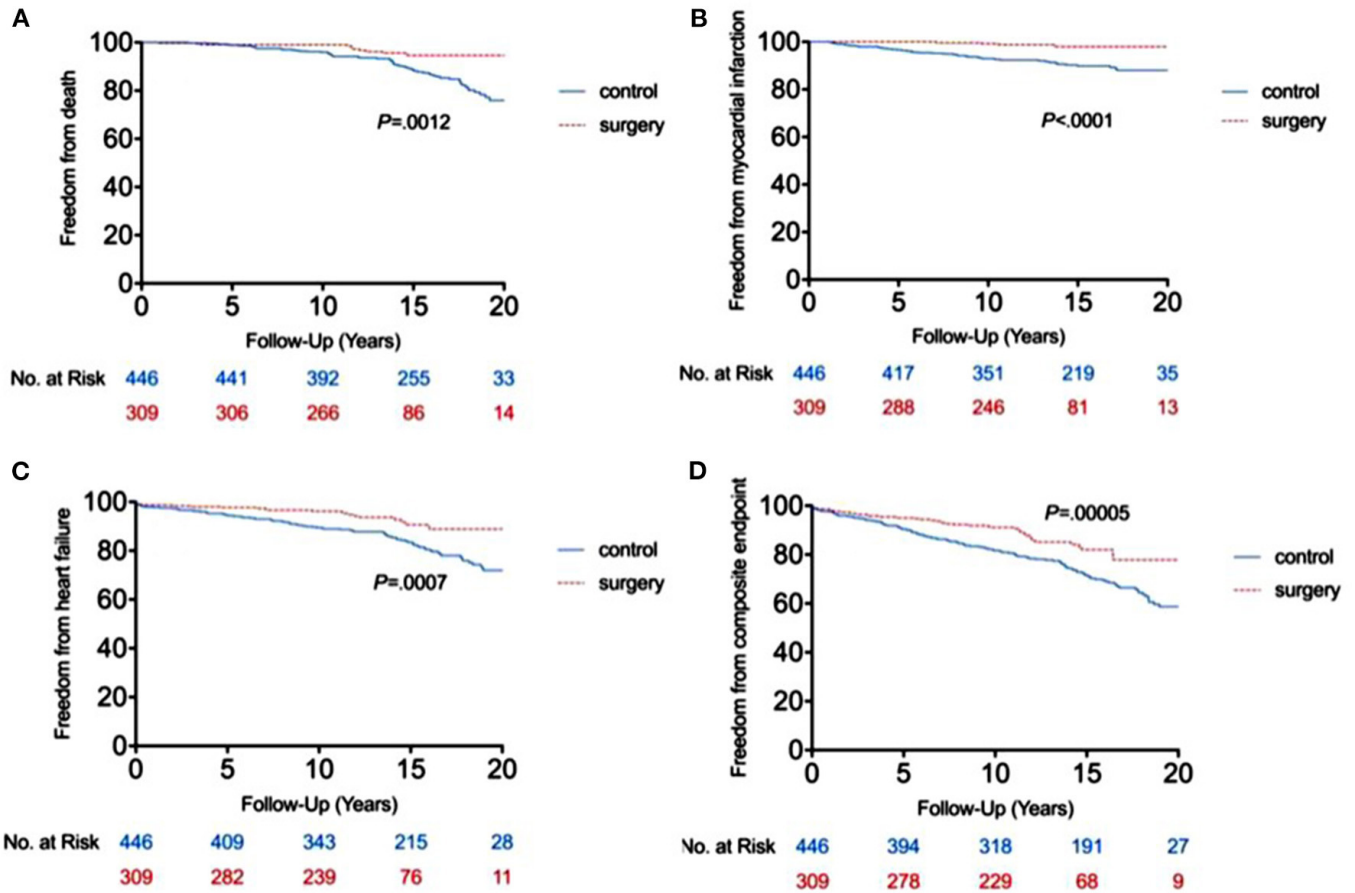
Freedom from end point events between RYGB-1Y group and NO-RYGB group. Kaplan-Meier curve showed the difference in mortality **(A)**, acute myocardial infarction **(B)**, heart failure admission **(C)** and composite events **(D)** in obese patients treated with early RYGB surgery within 1 year of index diagnosis (RYGB-1Y) compared with obese patients treated with medical only or later RYGB surgery 1 year after the index diagnosis (No-RYGB surgery).

The RYGB-1Y group had a lower risk of mortality (*P* = 0.001, log-rank test; Figure 2A), acute myocardial infarction (*P* < 0.001, log-rank test; Figure 2B), heart failure admission rate (*P* < 0.001, log-rank test; Figure 2C), and composite events (*P* < 0.001, log-rank test; Figure 2D) than the No-RYGB group. There was no difference in stroke rate. Univariate analysis is shown in Table 4. After adjusted for age and sex, multivariate analysis showed that RYGB-1Y group was independently associated with lower rates of mortality (hazard ratio, 0.51; 95% CI, 0.26–0.99; *P* = 0.04), acute MI (hazard ratio, 0.19; 95% CI, 0.07–0.53; *P* = 0.002), heart failure admission (hazard ratio, 0.60; 95% CI, 0.36–0.99; *P* = 0.04), and composite events (hazard ratio, 0.62; 95% CI, 0.44–0.88; *P* = 0.008; Table 4).

**Table 4:** Univariate and multivariate analysis on major adverse cardiovascular events.

	**Univariate analysis**			**Age and sex adjusted**		
**Variable**	**HR**	**95% CI**	***P*-value**	**HR**	**95% CI**	***P*-value**
Acute myocardial infarction	0.21	0.07–0.69	0.01	0.24	0.07–0.77	0.02
Heart failure admission	0.38	0.17–0.86	0.02	0.61	0.27–1.37	0.23
Stroke	1.00	0.53–1.91	0.99	1.23	0.64–2.35	0.54
Total mortality	0.24	0.07–0.78	0.02	0.31	0.10–1.02	0.05
Composite events	0.54	0.35–0.83	0.006	0.60	0.38–0.92	0.02

*HR, Hazard ratio; CI, Confidence interval*.

## Discussion

This large single-center study in the patient population from Olmsted County MN, USA, demonstrates that bariatric surgery within 1 year of index diagnosis and intensive medical management improves metabolic profile, cardiac event rate and survival. The markers of metabolic syndrome, including blood pressure, glucose level, and lipid levels, were substantially improved after surgery in patients with class II and III obesity, in parallel to a significant reduction in BMI. This favorable outcome was greater than those patients who received late or did not have bariatric surgery. Our primary and supplemental analysis did not show an association between RYGB surgery and lower risk of AF. However, early RYGB surgery MACE is beneficial in reduction of composite including acute myocardial infarction, stroke, heart failure admission, and in reduction of mortality.

### Bariatric Surgery and AF

Obesity is well-known to be an independent risk factor for AF (17, 18). Data from the Framingham Heart Study showed that obese patient had an overall 50% increase in the risk of AF compared with those of normal body weight (19). A study from our center showed a close relationship between BMI and progression of AF pattern from paroxysmal to permanent form (20). Obese patients are more likely to be symptomatic with AF, with lower quality-of-life scores, than lean and overweight groups (21). Pathophysiology studies also suggest a strong relationship among increased adiposity, impairment of atrial structure, and development of AF (22).

Recent studies suggested a long-term benefit from lifestyle modification and drug therapy for weight reduction in patients with atrial fibrillation. The ARREST-AF study included patients with a BMI ≥ 27 kg/m^2^ undergoing AF ablation and showed that AF frequency and duration were lower in the patients whose risk factors were actively managed when compared to controls (23). Prospective and randomized studies from the same institution found that long-term sustained weight loss was associated with a significant reduction of AF burden in the same group of obese individuals, irrespective of the type of therapy (24, 25). However, weight reduction is more challenging in the population with class II and III obesity with BMI ≥ 35 kg/m^2^. Surgical therapy is an effective approach in improving or preventing weight-related comorbidities, such as hypertension, type 2 diabetes mellitus, and obstructive sleep apnea in patients who have not responded to medical therapy (13–15, 26, 27). In our cohort, a substantial weight reduction and significant improvement in blood pressure, glucose and lipid control, were achieved in patients undergoing early surgical intervention, much greater than the late surgical or non-surgical patients.

Jamaly et al. reported in the Swedish population that when compared with usual care, weight loss through bariatric surgery reduced the risk of atrial fibrillation by 29%; this study had methodological difference in study design (28). The patient population was older and more often had hypertension than our study patients. Our study did not show a significant association between early RYGB surgery and development of AF. It is known that AF is aging associated epidemic disease. Approximately 1% of subjects at <60 years of age develop AF, whereas the AF incidence increases to 12% at 75–84 years of age (29). It is plausible that the surgical benefit for weight reduction in prevention of AF may not be seen till these patients become older when aging factor is confronted. As the majority of our study patients underwent RYGB were at early 40 of age, a longer follow-up duration may increase the power to support this observation. Furthermore, the vast majority of study patients were women who had 50% lower risk of developing AF than men within our follow-up period.

### Bariatric Surgery and Improvement in Metabolic Profile

We found that bariatric surgery was associated with a significantly greater improvement in cardiometabolic profiles in the RYGB-1Y group compared to No-RYGB-1Y group. In contrast, hemoglobin A1c level significantly decreased in the RYGB-1Y group while increased in the No-RYGB-1Y group, also suggesting better long-term glycemic management in early surgical patients. Similar findings were confirmed by Mingrone et al. in a recent randomized trial with 10-year follow-up, where participants randomized to RYGB had on average ~1% lower levels hemoglobin A1c level over follow-up when compared to the medical therapy group (30).

### Bariatric Surgery and Major Cardiovascular Events and Mortality

We observed significantly reduced risk of composite cardiovascular events by 46% as well as a significant reduction in the specific end point of myocardial infarction by 76%. Our finding concurred previous study that showed a similar composite cardiovascular risk reduction (31). Early RYGB surgery was associated with improved survival in our cohort. In one retrospective US-based cohort study, the long-term all-cause mortality of patients who underwent gastric bypass surgery was 40% lower than controls matched for age, sex, and BMI (32). The Swedish Obese Subjects study, a large, prospective matched surgical interventional trial, noted that patients who underwent bariatric surgery had a lower risk of mortality, with an adjusted hazard ratio of 0.71. Multiple other studies have demonstrated similar findings (33–35). Our study shows the survival benefit in patients who had early gastric bypass operation within 1 year of index diagnosis of AF, whereas other studies collected surgical patients at any time point after failing medical therapy. Early surgery at younger age with fewer comorbidities or medical complications may achieve a greater survival benefit from surgical approach.

### Study Limitations

Because this was a retrospective study, the bias of patient selection for surgery might have been introduced. Our patients did not receive regular cardiac rhythm monitoring; therefore, asymptomatic AF events may have been underdiagnosed, underestimating the true incidence of AF in this patient population. Furthermore, the small number of events may have limited the power to detect a significant association. Lastly, patients undergoing bariatric surgery might have been likely to have the AF identified in routine evaluations, as these patients tend a closer medical follow-up.

## Conclusion

RYGB surgery improves metabolic profile and is associated with lower cardiovascular events. However, the weight reduction from the surgery did not demonstrate a benefit for AF prevention in this young population. It is possible that longer follow-up duration may demonstrate a benefit of reduction in AF with bariatric surgery.

## Data Availability Statement

The raw data supporting the conclusions of this article could be made available by the corresponding author at a reasonable request.

## Ethics Statement

The studies involving human participants were reviewed and approved by Mayo Clinic Institutional Review Board. Written informed consent for participation was not required for this study in accordance with Minnesota statute and institutional requirements.

## Author Contributions

Y-MC, HY, JM-I, MC-C, FL-J, WM, YW, and YC: conceptualization. DH and KB: formal analysis. Y-MC, HY, JM-I, FL-J, WM, YW, MS, and YC: data curation. Y-MC, HY, JM-I, MC-C, and AC: writing—original draft preparation and writing—review and editing. Y-MC and YW: supervision. All authors have read and agreed to the published version of the manuscript and gave final approval and agree to be accountable for all aspects of work ensuring integrity and accuracy.

## Conflict of Interest

The authors declare that the research was conducted in the absence of any commercial or financial relationships that could be construed as a potential conflict of interest.
